# Hot-Pressed Super-Elastic Graphene Aerogel with Bidirectional Thermal Conduction Properties as Thermal Interface Materials

**DOI:** 10.3390/ma16237419

**Published:** 2023-11-29

**Authors:** Peng Lv, Xiaofeng Zhou, Songyue Chen

**Affiliations:** College of Electronic and Optical Engineering & College of Flexible Electronics (Future Technology), Nanjing University of Posts and Telecommunications, Nanjing 210023, China; xfzhou@njupt.edu.cn (X.Z.); sychen@njupt.edu.cn (S.C.)

**Keywords:** thermal interface materials, graphene, hot-pressing, thermal annealing, bidirectional

## Abstract

Traditional graphene-based films normally possess high thermal conductivity (TC) only along a single direction, which is not suitable for thermal interface materials (TIMs). Here, a graphene film with excellent bidirectional TC and mechanical properties was prepared by hot-pressing super-elastic graphene aerogel (SEGA). Thermal annealing at 1800 °C improves the further restacking of graphene sheets, bringing high structure stability to SEGA for enduring the hot-pressing process. The junctions and nodes between the graphene layers in the hot-pressed SEGA (HPSEGA) film provide bidirectional heat transport paths. The in-plane TC and through-plane TC of HPSEGA film with a thickness of 101 μm reach 740 Wm^−1^K^−1^ and 42.5 Wm^−1^K^−1^, respectively. In addition, HPSEGA film with higher thickness still maintains excellent thermal transport properties due to the interconnected structure reducing the effect of the defects. The infrared thermal images visually manifest the excellent thermal-transfer capability and thermal-dissipation efficiency of the HPSEGA films, indicating the great potential as advanced bidirectional TIMs.

## 1. Introduction

With the rapid development of 5G communication and artificial intelligence, high-power and highly integrated devices generate ultra-high heat-flow density during the operation, making the performance, lifetime, and reliability of electronics decline [1,2]. Thermal interface materials (TIMs) are widely used to bridge between the heat source and the heat sink to eliminate temperature [3,4,5]. Graphene with extraordinary intrinsic thermal conductivity (TC) of 3500–5300 Wm^−1^K^−1^ and excellent mechanical strength is regarded as one of the most potential TIMs [6,7].

To date, lots of research has focused on the highly thermal-conductive graphene films (GFs) normally assembled from graphene oxide (GO) sheets. GO film can be easily prepared by casting [8], vacuum filtration [9], electro-spray deposition [10], or evaporation-induced assembly [11] of GO aqueous solutions. Then the highly thermal-conductive GFs can be prepared by further reduction, carbonization, graphitization, and cold-compression of GO films [12]. During the preparation process, the graphene sheets tend to orientate and distribute in a single direction, especially under high pressure [13]. Sp^2^-hybridized carbon atoms allow for efficient phonon transport through the lattice oriented along the in-plane direction [14]. Thus, GFs composed of layer-by-layer stacked graphene sheets possess excellent in-plane TC (Table 1), which has a promising potential application as a high-performance heat spreader in thermal management systems [15]. However, the weak Van Der Waals coupling between the adjacent graphene sheets causes serious phonon scattering in the direction perpendicular to the lattice plane, leading to those conventional graphene films showing a through-plane TC 2–3 orders of magnitude lower than that in the in-plane direction [16,17,18]. To maximize the heat conduction efficiency of TIMs between the mating surface, they are required to possess not only high in-plane TC but also enhanced through-plane TC [19]. Some studies for the fabrication of vertically aligned structures of graphene have been reported [16,20,21,22,23,24], showing ultra-high through-plane TC (up to 615 Wm^−1^K^−1^) [25]. But they are still not suitable for TIMs, due to the sacrificed in-plane TC and high contact thermal resistance arising from the surface rigidity of vertical graphene [26].

The creation of a through-plane heat transfer pathway for forming the 3D thermal conductive network is considered as an efficient solution that could further extend the applications of GFs in the TIM field. Researchers attempted to introduce nanoparticles (such as Cu nanoparticles [27], Au nanoparticles [28], and SiO_2_@C nanoparticles [29]) or 1D nanomaterials (such as carbon nanotubes [17,30,31,32], SiC nanorod [33], and carbon nanoring [34]) into the interlayer of graphene sheets to enhance the heat conduction in the through-plane direction. However, the intercalation of nanoparticles inevitably induces decreased densities and increased micro-gaps, which leads to a decrease in in-plane TC. During the directional compression, 1D nanofillers tend to orient along the direction parallel to the graphitic lattice plane, leading to structure damage to the thermal transport bridge. Thus, there is still a challenge to design a novel structure of GFs with high in-plane TC and high through-plane TC.

**Table 1:** TC, thickness, and density of the highly thermal-conductive graphene-based films reported in references.

Highly Thermal-Conductive Graphene-Based Films	In-Plane TC (W m^−1^ K^−1^)	Through-Plane TC (W m^−1^ K^−1^)	Thickness (μm)	Density (g cm^−3^)
High TC in single direction				
Graphene paper [10]	1434	-	-	2.1
Graphene-based hybrid film [11]	1597	2.65	7	-
GF [35]	1940	-	10	2.03
GF [36]	1043.5	-	-	-
Ultra-thin GF [37]	3200	-	0.8	2.1
GF [38]	1100	-	8.1	
Large-sized GF [39]	803.1	3.98	14	2.05
Ball-milling exfoliated graphene paper [40]	1529	-	30	1.8
Glucose-modified GF [41]	1300	-	-	-
Electrochemically exfoliated graphene paper [42]	1022.8	-	-	-
Bidirectional high TC				
Vertical carbon nanotube@SiC-graphene film [17]	397.9	41.7	200	-
Graphene/nanocopper film [27]	234.9	5.22	-	-
Graphene-SiO_2_@C film [29]	36.54	6.65	-	-
Graphene–carbon nanotube [30]	933.37	6.27	106	0.985
Graphene–carbon nanotube–graphite film [31]	182.6	32.96	6000	1.67
Graphene hybrid paper [33]	263	17.6	500	0.8
Carbon nanoring/graphene hybrid paper [34]	890	5.81	-	-
This work	740.3	42.5	101	1.35
	688.1	39.6	192	1.57

In addition, according to Fourier’s law, both high TC and enlarged thickness (determines the cross-sectional area) are desirable in maximizing the heat transfer capability at the in-plane direction of the TIMs [13,30,43]. Nevertheless, the high TC is demonstrated only with extremely thin GFs of less than 30 μm (Table 1). As the thickness increases, the multiple grain boundary defects of the GFs increase, leading to the decline of TC. It less favored ultra-high thermal flux equipment (102–106 W cm^−2^) than metal-based TIMs [30]. The GFs with bidirectional superior thermal conduction performances and high heat flux have not been reported. Thus, there is still a major challenge to prepare highly thick GFs without sacrificing overall thermal properties.

**Figure 1:**
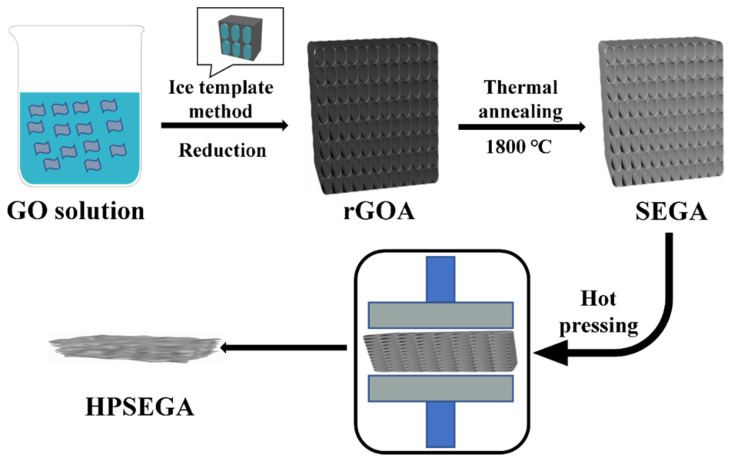
The schematic drawing of preparation of HPSEGA film.

Herein, the super-elastic graphene aerogels (SEGAs) were fabricated by ice template method and thermal annealing process (1800 °C). The SEGAs can maintain the continuously interconnected structure even at ultra-high compressive strain due to the thermal treatment improving the toughness of the cell walls assembled by the tightly stacked graphene sheets (Supplementary Material, Figure S1). Then the SEGAs were hot-pressed at 30–50 MPa and 1800 °C to form the hot-pressed SEGA (HPSEGA) films (Figure 1) with dense structure and interconnected morphology. In the HPSEGA, there are continuous thermal transport paths between adjacent graphene layers without any nanoparticles or 1D nanofillers, which eliminates the interface thermal resistance in the films [44]. In addition, the thick HPSEGA film with a thickness of up to 192 μm can be obtained by hot-pressing thick SEGA. Because of the low interface thermal resistance and dense structure, the HPSEGA thick film achieves excellent bidirectional thermal transport performances. The advanced thermal interfacial performances of the HPSEGA films are directly certified by thermal infrared imaging. Our work provides a novel way to prepare high-performance bidirectional GF TIMs with high heat flux.

## 2. Materials and Methods

### 2.1. Preparation of HPSEGA Film

Graphene oxide (GO) dispersion was synthesized by the modified Hummers method. The reduced GO aerogel (rGOA) was self-assembled by the ice template method according to the previous process [41]. In a typical process, GO aqueous solution with L-ascorbic acid was heated at 90 °C for 20 min to produce partially reduced GO hydrogel. The hydrogel was frozen in a refrigerator at −18 °C for 5 h and was subsequently thawed at room temperature for 2 h. A further reduction procedure was carried out for 8 h at 95 °C using initial L-ascorbic acid. After drying at 50 °C, the rGOA was prepared. For obtaining the SEGA, the rGOA was thermally annealed at 1800 °C under vacuum conditions to form tightly packed graphene cell walls with improved mechanical robustness. Subsequently, the HPSEGA films were obtained by hot-pressing the SEGAs using the high-temperature vacuum hot-pressing equipment (Shanghai Haoyue Furnace Technology Co., Ltd. VVPgr-40-2000, Shanghai, China). The SEGAs were placed into a high-strength graphite mold, and a pressure of 10 MPa was loaded on the mold. The whole hot-pressing process was carried out under the vacuum degree of 10^−2^ Pa to avoid the oxidation of the samples and the heating elements of the furnace. The heating and cooling progress is as follows: heating from room temperature up to 1400 °C with a rate of 9 °C min^−1^; then heating up to 1800 °C with a rate of 5 °C min^−1^; keeping 1800 °C for 1 h; finally cooling to room temperature with the help of water-cooling system. 

### 2.2. Characterization

The micro-morphology of the samples was observed by the field-emission scanning electron microscope (SEM, Hitachi S4800, Tokyo, Japan) system at 5 KV. The surface roughness of the films was recorded by atomic force microscopy (AFM, FM-Nanoview1000, VEECO, Suzhou, China). Compressive stress/strain measurements of SEGA and tensile test of HPSEGA films were carried out on the single-column system (Instron 5843, Norwood, MA, USA) with a 1 KN load cell. For compressive stress/strain measurement, the aerogel samples are cubes with a dimension of 30 mm × 30 mm (length × width), and the strain rate is 5% per second. For the tensile test, the film samples are rectangular in shape with a dimension of 5 mm × 10 mm (length × width), and the tensile rate is 1 mm s^−^^1^. The crystal structure was investigated by X-ray diffraction (XRD, D8 Advance, Bruker, Germany) using Cu Kα1 radiation (λ = 1.54 Å) with a scan rate of 4°/min. X-ray photoelectron spectroscopy (XPS, Physical Electronics, PHI-5300, Chanhassen, MN, USA) spectra were collected with a monochromatic Mg Ka radiation at a voltage of 14 kV and a power of 250 W. The in-plane and through-plane TC values of the films were calculated using the following formula:

(1) $$\kappa =\alpha \rho C_{p}$$

where κ, α, ρ, and *C_p_* are the TC, thermal diffusivity, density, and specific heat capacity of the films, respectively. The thermal diffusivity (α) was measured by a laser flash apparatus (LFA 467, Netzsch, Germany); the specific heat capacity (*C_p_*) was measured by a differential scanning calorimeter (Q20, TA instruments, New Castle, DE, USA); and the density (ρ) was measured by the water displacement method. The infrared thermal imager (Ti32, Fluke, Everett, WA, USA) was used to record the temperature distribution images of the samples.

## 3. Results and Discussion

**Figure 2:**
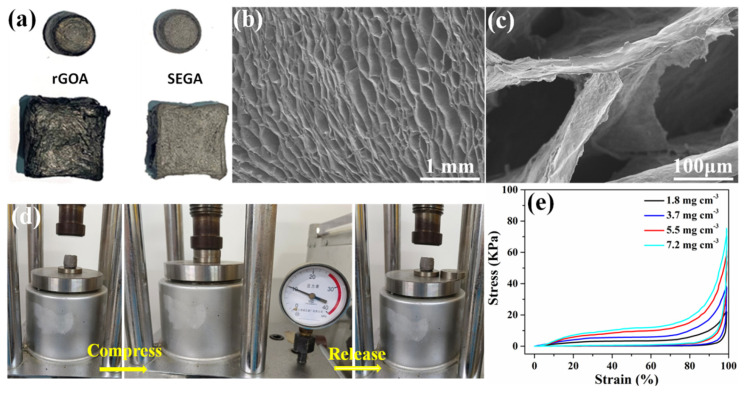
(**a**) Digital images of rGOAs and SEGAs, respectively; (**b**,**c**) SEM images of a cross–section of SEGA; (**d**) real–time photos of the compression-release process of SEGA; (**e**) compressible stress–strain curves of SEGAs with various densities.

Figure 2a displays the rGOAs and SEGAs, respectively. The shape and size of the aerogels depend on the utilized reactor during the ice-template process. It can be found that, after the 1800 °C thermal annealing process, the color of the aerogels turns from black to silvery metallic luster, indicating the full repair of defects of rGO sheets. Figure 2b shows the microstructure of the SEGAs at the cross-section view, which presents oriented cellular and honeycomb-like architecture. Each cell wall is composed of tightly stacked graphene sheets and converges at the junctions or nodes (Figure 2c). The SEGAs with a 3D interconnected network are significantly different from the rGO films with parallel structures used for preparing the highly thermal-conductive GF films. The interconnected structure of the cell walls is in favor of reducing the interface contact thermal resistance in the aerogels and providing a continuous thermal transport path along the network. As shown in Figure 2d, the SEGA was squeezed into slices under a pressure of 10 MPa. And SEGA can completely recover to its original shape rapidly once the external pressure is removed. In contrast, the rGOA deforms permanently indicating a structural collapse (Figure S2). SEGAs with various densities (1.8~7.2 mg cm^−3^) were prepared by controlling the GO concentrations (Figure S3e). As shown in Figure S3a–d, with the increasing concentration of GO solution, the average pore size becomes smaller and the porous structure turns denser, while the oriented cellar structure of SEGA preserves well. The strain/stress curves of the SEGAs with various densities are shown in Figure 2e, indicating a high recoverable compressive strain of 99%. The strain–stress curves for 100 cycles (Figure S3f) indicate the structure stabilization of SEGA. Higher compressive strain (>99%) of the sample cannot be measured accurately due to the limitation of the equipment sensitivity. The high compressibility arises from the regular cellular structure and mechanical robustness of tightly packed cell walls in SEGAs. This ultra-high structure stability is crucial for preparing GF films with interlayer junction structure and bidirectional heat transport path even after the hot-pressing process.

**Figure 3:**
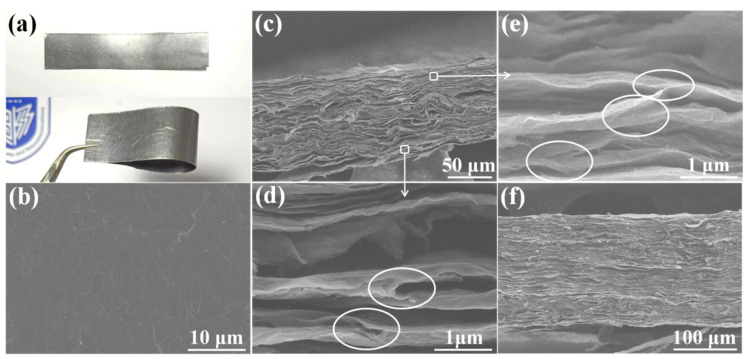
(**a**) Digital image of HPSEGA films; (**b**) SEM image of the surface of HPEGA film; cross-sectional SEM morphology of the HPSEGA thin film (**c**–**e**) and the HPSEGA thick film (**f**).

As shown in Figure 3a, the HPSEGA films prepared by hot-pressing SEGA are smooth, flexible, and uniform with a silvery metallic luster. The microstructure of the surface of HPSEGA film shows a completely continuous surface (Figure 3b and Figure S4). And the microstructure of the edge of the HPSEGA film has been discussed (Figure S5). As shown in the cross-sectional SEM in Figure 3c, a HPSEGA thin film shows a thickness of 101 μm, which is prepared by hot-pressing a 2 cm thick SEGA with a density of 7.2 mg cm^−3^. The density of this HPSEGA film reaches 1.35 g cm^−3^. It can be found that the graphene layers tend to orient perpendicularly to the pressure direction, which provides the heat pathway for in-plane phonon transports. High-magnification SEM images indicate that the junctions and nodes between the adjacent graphene layers still maintain well even after the hot-pressing process (marked with circles in Figure 3d,e). This interconnected structure is significantly different from the typical layered structure of the traditional GFs prepared from the GO films (Figure S6). Those junctions and nodes in HPSEGA films act as the “bridges” to connect adjacent graphene sheets in the cross-plane direction, which can effectively enhance the through-plane TC. In addition, a thicker HPSEGA film (192 μm) exhibited a similar structure (Figure 3f) can be obtained by hot-pressing thicker SEGA (4 cm). This thicker film possesses a higher density (1.57 g cm^−3^) than that of the thinner HPSEGA film (101 μm), which may be attributed to a more effective stress load for thicker SEGA during the hot-pressing process. We envision that HPSEGA films with higher thickness can be achieved by just increasing the thickness of the SEGAs.

**Figure 4:**
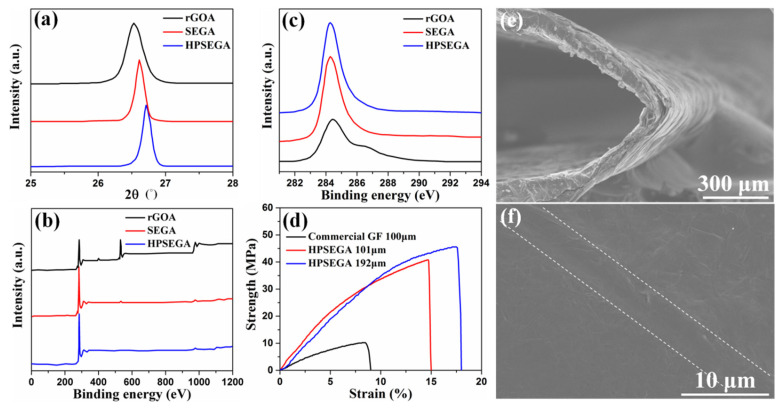
(**a**) XRD spectra, (**b**) XPS spectra, and (**c**) C1s of the rGOA, SEGA, and HPSEGA films, respectively; (**d**) stress–strain curves of the films; SEM images of (**e**) cross-section view of HPSEGA film under 135° bending and (**f**) surface morphology change in HPSEGA film after 10 cycles of bending/releasing.

XRD and XPS were used to prove the deoxygenation and interlaminar consolidation of samples during the fabrication process. As shown in XRD patterns in Figure 4a, rGOA, SEGA, and HPSEGA films show the typical peaks at the 2θ value of 26.53°, 26.62°, and 26.71° corresponding to the d-spacing of 3.53 Å, 3.45 Å, and 3.36 Å. The reducing interlayer distance is attributed to the thermal reduction (annealing at 1800 °C), which removes oxide-containing groups, repairs the structural defects, and induces the structural transformation from incommensurate stacking to AB stacking. In addition, based on the “stress graphitization” mechanism, the combination of heating and pressing promotes graphitic transformation, repairing defects, increasing the mean size of ordered stacking, and decreasing the interlayer spacing. As shown in the XPS spectra (Figure 4b), the O1s peaks of SEGA and HPSEGA film become negligible, and the carbon to oxygen (C/O) atomic ratio of rGOA is 4.66 and promotes remarkably to 71.21 and 76.43 for SEGA and HPSEGA film, confirming an almost complete removing of oxide-containing groups after thermal annealing. The pronounced and sharp C=C/C-C peaks of SEGA and HPSEGA in Figure 4c indicate the remarkable restoration of the sp^2^-bonded carbon lattice structure. 

The mechanical performances of the HPSEGA films have been investigated. A commercially available GF (Hefei AOQI Electronic Technology Co., Ltd., Hefei, China, GSH-T100, 100 μm thick) was also tested for comparison. Figure 4d and Figure S7 show the stress–strain curves of the films under tensile stress. HPSGA film with a thickness of 101 μm shows a strain of 15% under 39.8 MPa of tensile stress. At a given strain value, the HPSEGA films can endure about 3 times larger stress compared with the commercial GF. And the HPSEGA film possesses higher tensile stress and strain. The breaking strength of the HPSEGA films prepared by the SEGAs with various densities is shown in Table S1. It can be found that the tensile strength of HPSEGA films increases with the increased density of the SEGAs. As shown in Figure S3, SEGAs with higher density possess more junctions and nodes. After the hot-pressing process, these junctions and nodes in the HPSEGA films can load higher stress. To test the flexible property of HPSEGA film, a bending test was performed (Figure S8). From Figure 4e, HPSEGA film can maintain structure stability under 135° bending. It exhibits excellent flexibility and reliability after 10 cycles of bending/releasing with slight wrinkles (Figure 4f). In comparison, significant breakages appear on the surface of commercial GF (Figure S9).

This strong mechanical property of HPSEGA is attributed to the interconnected cross-linked structure between graphene layers. This special structure can maintain well even under hot-pressing is attributed to two factors: 1. Thermal annealing of SEGA at 1800 °C improves the π-π interaction between the graphene sheets in the cell walls, leading to larger mean size of ordered stacking of graphene and imparting high toughness and mechanical robustness to the cell walls. Thus, SEGA possesses a high structure stability to bear the hot-pressing process. 2. In the previous literature, the thermal annealing process (2600–2800 °C) and the cold-pressing process (50–300 MPa) were carried out individually to prepare GFs [12,13,37,39,42]. According to the theory of “stress graphitization” [45], the pressure-induced effect accelerates the transformation of incommensurately stacked carbon to graphene. Thus, lower pressure (10 MPa) and lower annealing temperature (1800 °C) are needed during the hot-pressing process in this work. And the relatively low pressure and temperature are in favor of maintaining the interconnected structure between graphene layers during the preparation process.

**Figure 5:**
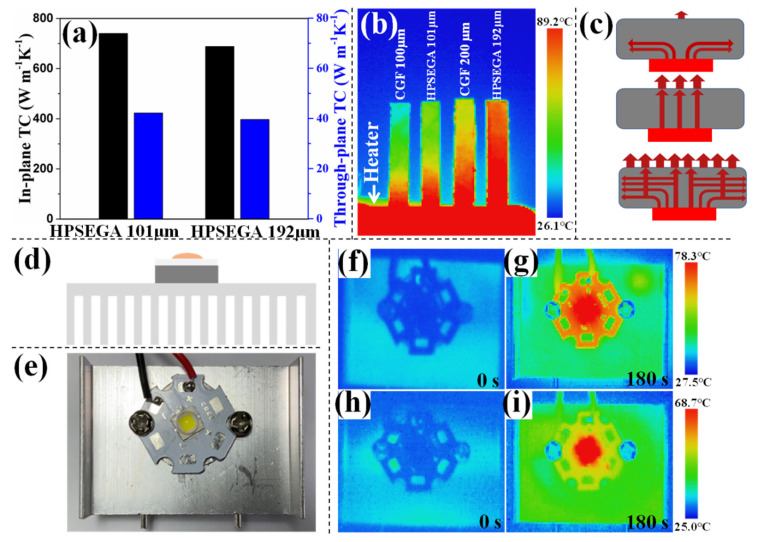
(**a**) TC values of the HPSEGA films; (**b**) infrared thermal image of the films attached vertically on a constant temperature heat source; (**c**) schematic diagram of the thermal transport mechanism in GF with a horizontal structure, GF with vertical structure, and HPSEGA film; (**d**) schematic diagram and (**e**) photos of the LED devices, in which the films are mounted between the LED and heat sink; (**f**–**i**) infrared thermal images showing the heat dissipation of the LED lamp with (**f**,**g**) commercial GF and (**h**,**i**) HPSEGA film as TIMs. The images were captured at (**f**,**h**) 0 s and (**g**,**i**) 180 s.

The TC values of HPSEGA films have been measured [46,47]. The *C_p_* is measured as 0.765 Jg^−1^K^−1^. As shown in Figure 5a, HPSEGA film with a thickness of 101 μm and prepared by the SEGA with a density of 7.2 mg cm^−3^ exhibits an in-plane TC of 740.3 Wm^−1^K^−1^ and through-plane TC of 42.5 Wm^−1^K^−1^. In addition, as shown in Table S1, HPSEGA films prepared by the SEGAs with higher density processes exhibit higher TC values, arising from more junctions and nodes, providing more heat transport pathways. In comparison with the GFs listed in Table 1, especially those with high bidirectional TC values, HPSEGA films show an excellent combination of thermal transport capabilities. For example, compared with graphene/carbon nanotube film [30], HPESGA film with a similar thickness (~100 μm) possesses a relatively low in-plane TC, but its through-plane TC is ~7 times higher than that of graphene/carbon nanotube film. HPSEGA film with a thickness of 192 μm shows slightly lower through-plane TC (39.6 Wm^−1^K^−1^) than that of vertical carbon nanotube@SiC-graphene hybrid film [17], whereas its in-plane TC (688.1 Wm^−1^K^−1^) is 1.7 times higher than that of the hybrid film. The HPSEGA film achieves a superior bidirectional thermal transport mechanism as follows. Firstly, the graphene sheets are arranged horizontally and play a main role in phonon transport in the in-plane direction, which leads to high in-plane TC values. Secondly, the junctions and nodes connected to the adjacent graphene layers in the cross-plane direction contribute to the pathway for through-plane phonon transport. As mentioned above, normally the TC values of GFs decline significantly with the thickness enlargement due to the increase in interfaces. However, HPSEGA film with higher thickness (191 μm) still shows high TC values in both in-plane direction and through-plane direction, arising from the interconnected structure providing more heat path and reducing the effect of the interface in the film. 

The heat transfer capability of the thermal conductive films was characterized by infrared thermal images (Figure 5b). The commercial thin GF (100 μm, in-plane TC = 800 Wm^−1^K^−1^, through-plane TC = 10 Wm^−1^K^−1^), commercial thick GF (200 μm, in-plane TC = 500 Wm^−1^K^−1^, through-plane TC = 5 Wm^−1^K^−1^), HPSEGA film (101 μm), and HPSEGA film (192 μm) were caught in the middle of a constant temperature heat source. It is obvious that the thick films show a more uniform temperature distribution, even though the thin films possess higher TC. It indicates that thickness is crucial to the heat flux for thermally conductive material in practical applications. In addition, the brighter top-end image of HPEAG film (192 μm) means higher surface temperature and indicates higher heat-transfer efficiency in comparison with the commercial thick GF (200 μm), which is attributed to both high TC values and thickness. 

Figure 5c presents a schematic of different GFs as the TIMs. In horizontal GF and vertical GF, the aligned graphene array provides a heat transport pathway only along a single direction. None of them are not ideal TIMs. But HPSEGA film with bidirectional thermal conductivity can significantly enhance the heat-dissipation effect between the heat source and the heat sink. Utilizing the highly bidirectional TC of HPSEGA, we demonstrated its use as a TIM for heat dissipation of a high-power LED lamp (Figure 5d,e). For comparison, commercial GF was also used (Figure 5h,i). The films were mounted between a high-power LED and an aluminum heat sink (Figure 5e). The temperature change is monitored by the infrared thermal imager. It can be found that the temperatures of the LED with HPSEGA film (101 μm) as TIM are 68.7 °C and 25.0 °C lower than that with commercial GF (192 μm) after 180 s operation (Figure 5f–i). The long-term heat-dissipation performance tests confirm the excellent durability and stability of the HPSEGA films (Figure S10). These results demonstrate the great potential of the HPSEGA film for TIMs in thermal management systems.

## 4. Conclusions

In summary, the HPSEGA film with both high in-plane TC value and through-plane TC value was prepared by hot-pressing the SEGA. The structure characterization results indicate that the thermal annealing process improves the further restacking of graphene sheets in cell walls, bringing high structure stability to the SEGA during the hot-pressing process. Micro-morphology characterization shows that the junctions and nodes between graphene layers can be maintained in HPSEGA film, which provides the heat transport path in both in-plane direction and through-plane direction. The HPSEGA films possess excellent bidirectional thermal transport ability. The in-plane TC value and through-plane TC value of HPSEGA film (101 μm) reach 740.3 Wm^−1^K^−1^ and 42.5 Wm^−1^K^−1^, respectively. Infrared thermal images visually manifest the advanced heat-dissipation efficiency of HPSEGA film as TIMs compared with the commercial GF. In addition, HPSEGA film with higher thickness (200 μm) also possesses high bidirectional TC values due to its interconnected structure. By getting rid of the aging issue of conventional GF TIMs, the HPSEGA film with interconnected structure has promising potential to be applied as high-performance TIMs with good mechanical and thermal properties.

## Data Availability

Data are contained within the article.

## Supplementary Materials

The following supporting information can be downloaded at: https://www.mdpi.com/article/10.3390/ma16237419/s1, Figure S1: Schematic diagram of a cell in SEGAs; Figure S2: Real-time photos of the compression-recovery process of rGOA; Figure S3: SEGAs prepared with GO concentration of (a) 2 mg mL^−1^, (b) 4 mg mL^−1^, (c) 6 mg mL^−1^, (d) 8 mg mL^−1^, (e) Plot of the density of SEGAs versus the GO concentration, (f) Compressive stress of SEGA with density of 7.2 mg cm^−2^ at strain of 99% for 100 compress/release cycles; Figure S4: AFM images of the surface of (a) rGO film, (b) HPSEGA film; Figure S5: (a) Photos of SEGAs, (b) surface SEM images of the SEGAs, SEM images of the edge of (c) the HPSEGA film and (d) the traditional graphene film, (e) Schematic diagram of the edge structure of the HPSEGA film; Figure S6: Cross-sectional SEM images of GFs prepared from GO films; Figure S7: Stress-strain curves of HPSEGA films; Figure S8: Real-time photos of the folding-recovery process of HPSEGA film; Figure S9: SEM images of surface change of commercial GF after bending test; Figure S10: Infrared thermal images of LED lamp with HPSEGA film as TIMs captured at (a) 0 s, (b) 180 s, and (c) 72 h; Table S1: Tensile strength and TC values of HPSEGA films prepared by the SEGAs with various densities [37,48].
