# Supplementary material for: Hot-Pressed Super-Elastic Graphene Aerogel with Bidirectional Thermal Conduction Properties as Thermal Interface Materials
Source: Materials (Basel). 2023 Nov 29;16(23):7419. doi: 10.3390/ma16237419 (PMC10706873; doi:10.3390/ma16237419)
Supplement: Supplementary file 1 [file materials-16-07419-s001.zip › materials-2725504-supplementary.pdf]

materials

Article

Hot-pressed super-elastic graphene aerogel with bidirectional thermal conduction properties as thermal interface materials

Peng Lv, Xiaofeng Zhou and Songyue Chen

College of Electronic and Optical Engineering & College of Flexible Electronics (Future Technology), Nanjing University of Posts and Telecommunications, Nanjing 210023, China

Preparation of GO:

GO was synthesized from natural graphite (325 mesh) via a modified Hummers' method. Briefly, 1g graphite powder, 3g $KMnO_4$, and 60mL concentrated H_2SO_4 solution without mix were respectively introduced in a refrigerator (-18°C) for 30 min. Then the graphite powder, $KMnO_4$, and concentrated H_2SO_4 solution were successively introduced in a flask (250mL). The mixture was stirred for 2h and the temperature was controlled below 20°C in an ice bath. After these steps, the mixture was moved to an oil bath at 50°C and sequentially stirred for 6 hours. The resulting mixture was slowly poured into a 500mL beaker with 120mL of distilled water. The 30% H_2O_2 solution was dropwise added into the mixture until no bubbling. Finally, the crude product was washed repeatedly with deionized water by centrifuge until the filtrate became neutral. The obtained brown-yellow graphene oxide was saved for use.

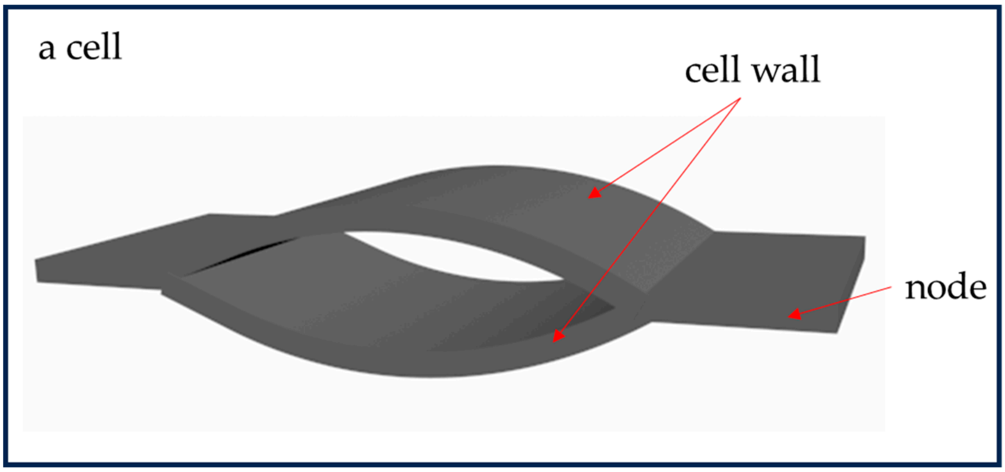

Figure S1 Schematic diagram of a cell in SEGAs.

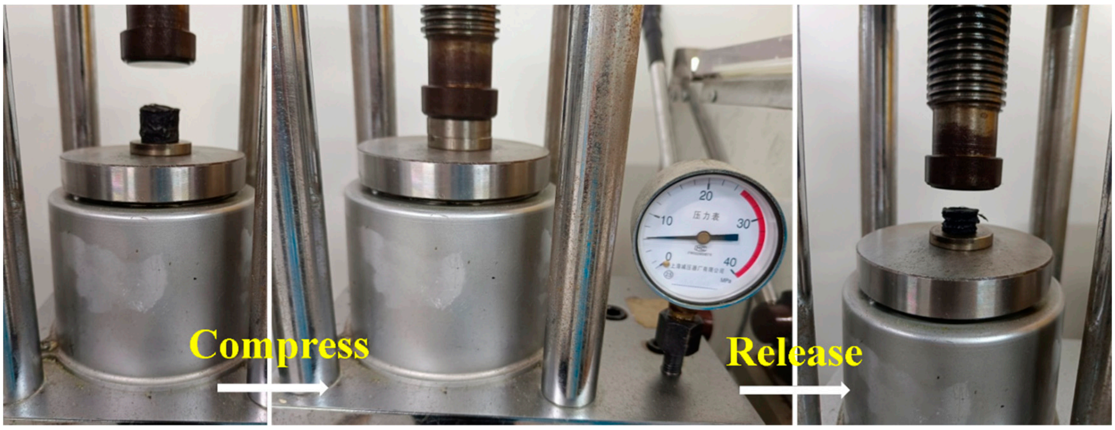

Figure S2 Real-time photos of the compression-recovery process of rGOA.

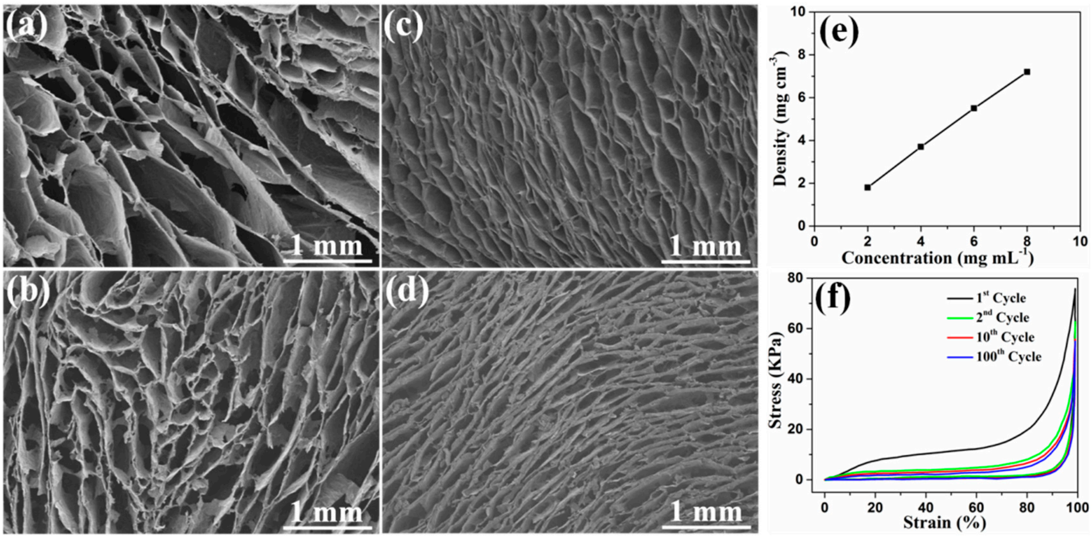

Figure S3 SEGAs prepared with GO concentration of (a) 2 mg mL^{-1}, (b) 4 mg mL^{-1}, (c) 6 mg mL^{-1}, (d) 8 mg mL^{-1}; (e) Plot of the density of SEGAs versus the GO concentration; (f) Compressive stress of SEGA with density of 7.2 mg cm^{-2} at strain of 99% for 100 compress/release cycles.

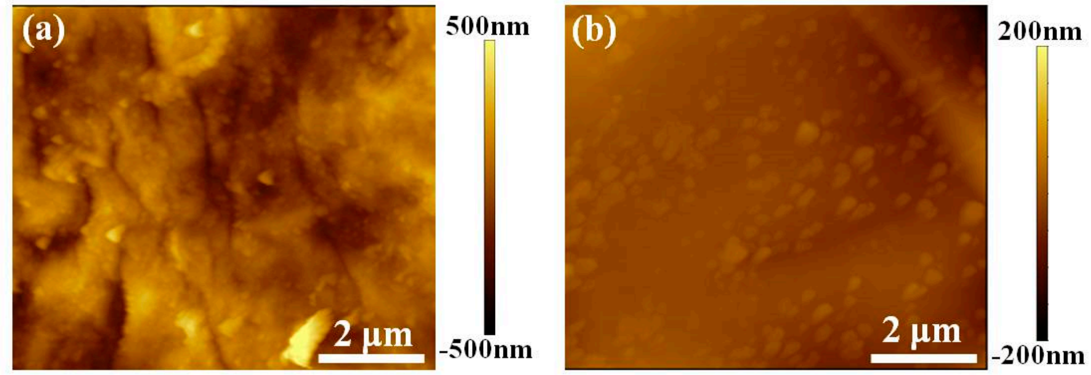

Figure S4 AFM images of the surface of (a) rGO film and (b) HPSEGA film.

The surface morphology of the films was recorded by the atomic force microscopy (AFM, FM-Nanoview1000, VEECO, China). For comparison, a rGO slice was peeled off from the rGOA to characterize by AFM too. As shown in Figure S4a, the rGO film exhibited a relatively rough topography with small smooth features. We assume that the small smooth features were caused by the wrinkle nature of rGO sheets and the random overlapping of individual rGO sheets during the self-assembly process. For the HPSEGA film, larger smooth features and a flat topography were observed (Figure S4b). This shows that the hot-pressing process can lead to the coalescence of adjacent graphene sheets and transform the originally random stacked GO sheets to flat and extended graphene layers [37,48].

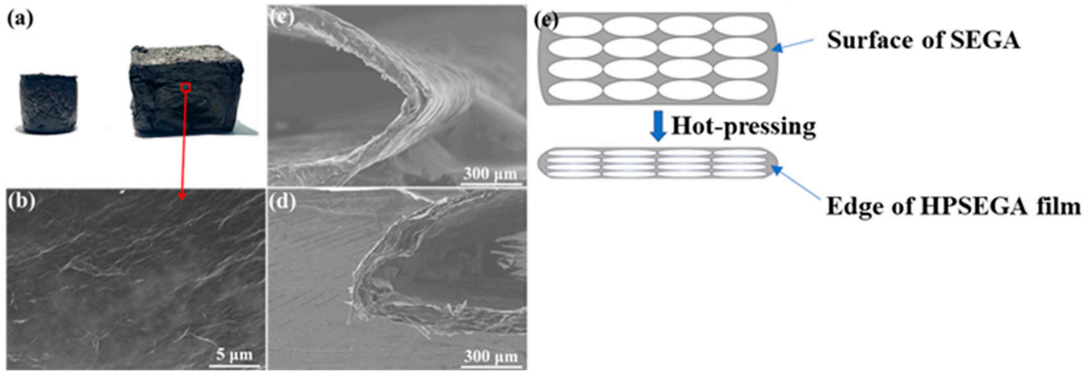

Figure S5 (a) Photos of SEGAs; (b) surface SEM images of the SEGAs; SEM images of the edge of (c) the HPSEGA film and (d) the traditional graphene film; (e) Schematic diagram of the edge structure of the HPSEGA film.

The surface of SEGAs (Figure S5a,b) shows a continuous structure like a film, which is significantly different from the porous structure of the interior zone of SEGAs (Figure 2b). After hot-pressing process, the surface of SEGAs transform to the surface and the edge of the HPSEGA film. As shown in Figure S5c, the edge of the HPSEGA film is smooth, which is significantly different from the edge of the traditional graphene film with a multilayered structure (Figure S5d) and is also different from the inner zone of the HPSEGA film (Figure 3c, f). Figure S5e shows schematic diagram of the transformation from surface of SEGA to edge of HPSEA film.

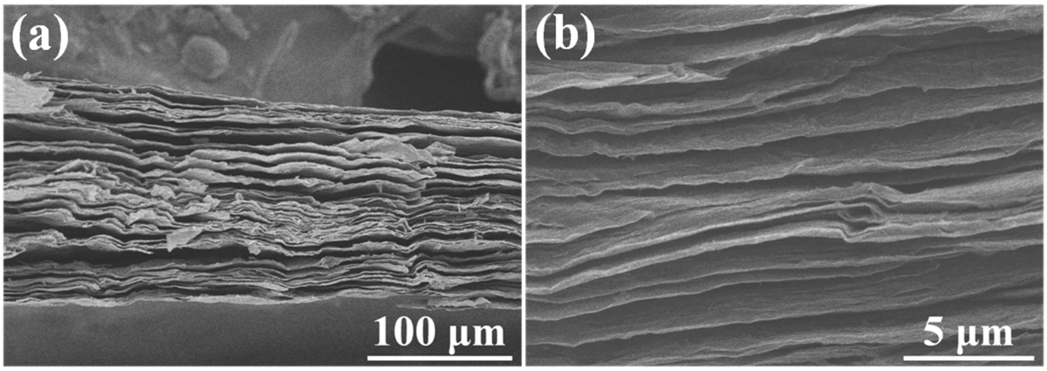

Figure S6 Cross-sectional SEM images of GFs prepared from GO films.

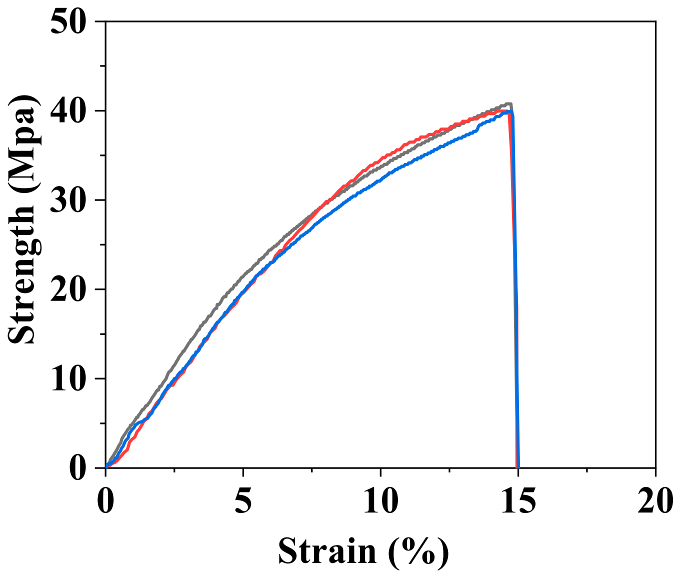

Figure S7 Stress-strain curves of HPSEGA films.

All the tensile tests have been carried out at least 3 times. As shown in the Figure S7, HPSEGA films show a high repeatability and reliability of mechanical performance.

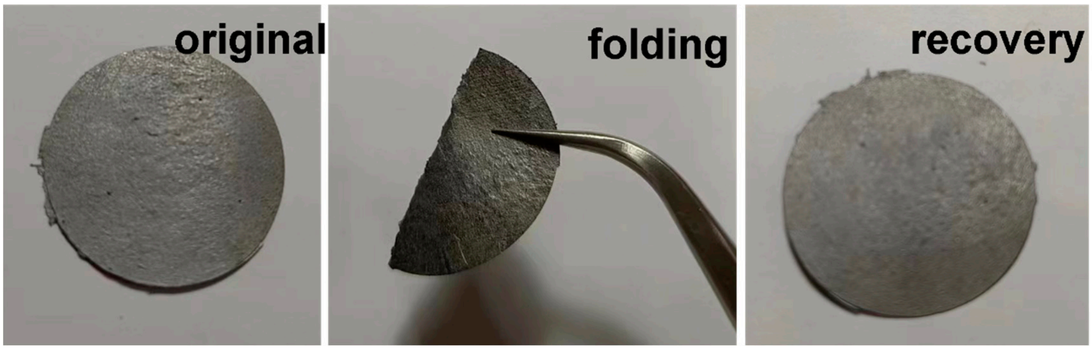

Figure S8 Real-time photos of the folding-recovery process of HPSEGA film.

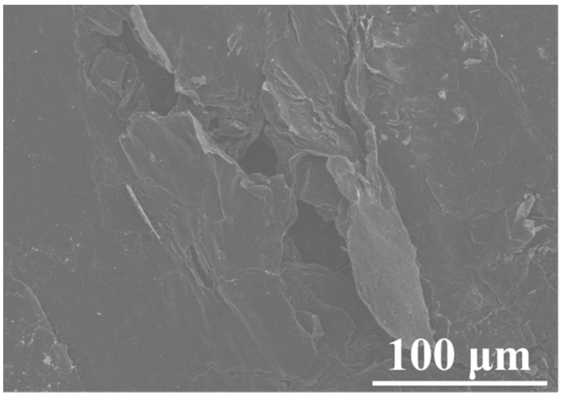

Figure S9 SEM images of surface change of commercial GF after bending test.

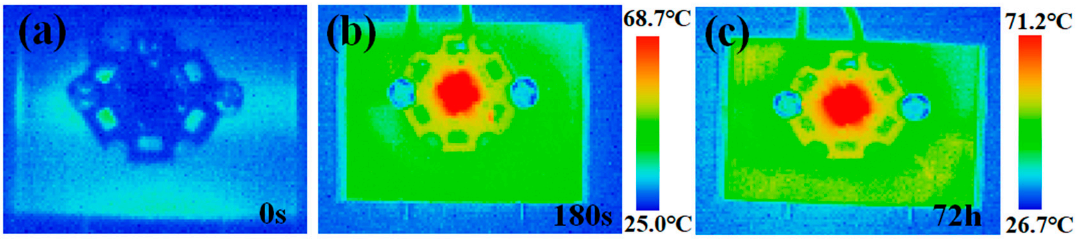

Figure S10 Infrared thermal images of LED lamp with HPSEGA film as TIMs captured at (a) 0s, (b)180s, and (c)72h.

Since the HPSEGA films are all-carbon materials, they possess high thermal stability at the temperature <400°C, which is different from the silicone thermal grease and the conductive polymer composites, where the thermal conductivity will degrade during long-term heat dissipation. The infrared thermal images of LED lamp for 72 h operation have been provided in the supporting information (Figure S10), which shows high durability and stability.

Table S1. Tensile strength and TC values of HPSEGA films prepared by the SEGAs with various densities.

Density of SEGAs (mg cm^{-3})	Density of HPSEGA film (mg cm^{-3})	Thickness of HPSEGA film (μm)	Breaking strength (MPa)	In-plane TC (W $m^{-1}k^{-1}$)	Through-plane TC (W $m^{-1}k^{-1}$)
1.8	0.89	96	33.8	441.2	24.4
3.7	1.13	98	36.2	512.7	31.5
5.5	1.28	99	37.4	605.3	36.2
7.2	1.35	101	39.8	740.3	42.5

References:

37. Wang, N.; Samani, M.K.; Li, H.; Dong, L.; Zhang, Z.; Su, P.; Chen, S.; Chen, J.; Huang, S.; Yuan, G.; et al. Tailoring the Thermal and Mechanical Properties of Graphene Film by Structural Engineering. *Small* **2018**, *14*, 1801346; https://doi.org/10.1002/smll.201801346.

48. Rozada, R.; Paredes, J.I.; Villar-Rodil, S.; Martínez-Alonso, A.; Tascón, J.M.D. Towards full repair of defects in reduced graphene oxide films by two-step graphitization. *Nano Research* **2013**, *6*, 216-233; https://doi.org/10.1007/s12274-013-0298-6.
